# Emergence of Tn*1999.7*, a New Transposon in *bla*_OXA-48_-Harboring Plasmids Associated with Increased Plasmid Stability

**DOI:** 10.1128/aac.00787-22

**Published:** 2022-10-06

**Authors:** Janko Sattler, Tsvetan Tsvetkov, Yvonne Stelzer, Sina Schäfer, Julian Sommer, Janina Noster, Stephan Göttig, Axel Hamprecht

**Affiliations:** a Institute for Medical Microbiology, Immunology and Hygiene, University Hospital Cologne and Faculty of Medicine, University of Cologne, Cologne, Germany; b DZIF (German Centre for Infection Research), Partner Site Bonn-Cologne, Cologne, Germany; c Institute for Medical Microbiology and Virology, University of Oldenburg and Klinikum Oldenburg, Oldenburg, Germany; d Institute for Medical Microbiology and Infection Control, Hospital of Johann Wolfgang Goethe University, Frankfurt, Germany

**Keywords:** OXA-48, Tn*1999*, carbapenemase, conjugation, horizontal gene transfer, plasmid stability, transposon

## Abstract

OXA-48 is the most common carbapenemase in *Enterobacterales* in Germany and many other European countries. Depending on the genomic location of *bla*_OXA-48_, OXA-48-producing isolates vary in phenotype and intra- and interspecies transferability of *bla*_OXA-48_. In most bacterial isolates, *bla*_OXA-48_ is located on one of seven variants of Tn*1999* (Tn*1999.1* to Tn*1999.6* and invTn*1999.2*). Here, a novel Tn*1999* variant, Tn*1999.7*, is described, which was identified in 11 clinical isolates from 2016 to 2020. Tn*1999.7* differs from Tn*1999.1* by the insertion of the 8,349-bp Tn*3* family transposon Tn*7442* between the *lysR* gene and *bla*_OXA-48_ open reading frame. Tn*7442* carries genes coding for a restriction endonuclease and a DNA methyltransferase as cargo, forming a type III restriction modification system. Tn*1999.7* was carried on an ~71-kb IncL plasmid in 9/11 isolates. In one isolate, Tn*1999.7* was situated on an ~76-kb plasmid, harboring an additional insertion sequence in the plasmid backbone. In one isolate, the plasmid size is only ~63 kb due to a deletion adjacent to Tn*7442* that extends into the plasmid backbone. Mean conjugation rates of the Tn*1999.7*-harboring plasmids in J53 ranged from 4.47 × 10^−5^ to 2.03 × 10^−2^, similar to conjugation rates of other pOXA-48-type IncL plasmids. The stability of plasmids with Tn*1999.7* was significantly higher than that of a Tn*1999.2*-harboring plasmid *in vitro*. This increase in stability could be related to the insertion of a restriction-modification system, which can promote postsegregational killing. The increased plasmid stability associated with Tn*1999.7* could contribute to the further spread of OXA-48.

## INTRODUCTION

**FIG 1:**
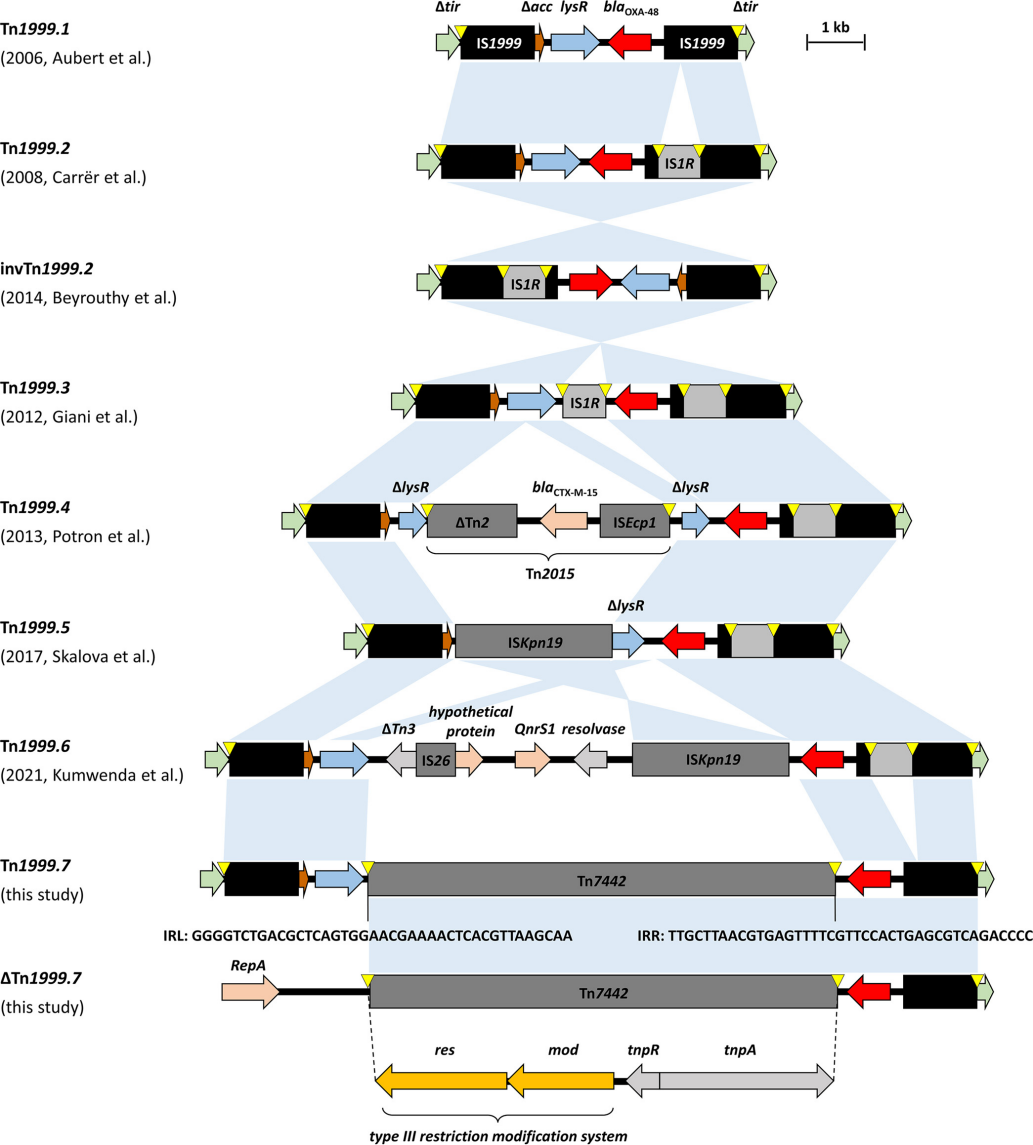
Overview of Tn*1999* variants located on pOXA-48-type plasmids (5–7, 42–45). Arrows indicate coding sequences and their directions: green arrows, Δ*tir*; brown arrows, Δ*aac* (acetyl-CoA carboxylase); blue arrows, (Δ)*lysR*; red arrows, bla_OXA-48_; gray arrows, genes related to mobile genetic elements; orange arrows, genes related to a restriction modification system; light red arrows, other genes. Gray and black boxes, insertion sequences and transposons; yellow triangles, target site duplications; IRL/IRR, inverted repeat left/right. The figure style was adapted from Pitout et al. (1).

The prevalence of carbapenemase-producing *Enterobacterales* (CPE) is increasing worldwide due to dissemination of CPE clones and the spread of carbapenemase genes via mobile genetic elements. OXA-48 carbapenemases are the most frequently detected carbapenemases in Western Europe, including Germany, North Africa, and the Middle East (1). The spread of OXA-48 is mainly linked to the dissemination of a highly conjugative 63.6-kb IncL plasmid (2), referred to as pOXA-48. Within this plasmid, *bla*_OXA-48_ is located between two copies of IS*1999* in variants of the composite transposon Tn*1999* (Fig. 1). As the transfer inhibition gene *tir* is disrupted by the insertion of Tn*1999*, pOXA-48 plasmids exhibit increased conjugation rates, which contributes to the spread of OXA-48 CPE (3). The most frequently identified Tn*1999* variant is Tn*1999.2*, followed by Tn*1999.1* and invTn*1999.2*, whereas the other variants are rarely detected (4). The transposon type can influence the phenotype of the host strain as well as mobilization characteristics of *bla*_OXA-48_. For example, some transposon variants alter the antibiotic resistance phenotype of the isolate by the insertion of another β-lactamase, as described for Tn*1999.4* (5), or increased expression of *bla*_OXA-48_ by modification of the promoter region, as described for Tn*1999.2* (6). On the other hand, invTn*1999.2* has been associated with chromosomal integration of *bla*_OXA-48_ (7).

This study analyzed the genetic background of a novel Tn*1999* variant, termed Tn*1999.*7, identified in clinical isolates of different OXA-48-producing *Enterobacterales* species. Furthermore, we aimed to investigate possible influences of Tn*1999.7* on conjugation and plasmid stability, as well as the resistance phenotype of the host strain.

## RESULTS AND DISCUSSION

### Epidemiology.

Between 2013 and 2020, 139 OXA-48-like CPE were collected at the University Hospital Cologne. After removing isolates with non-OXA-48 carbapenemases (e.g., OXA-181) and with *bla*_OXA-48_ not located on IncL plasmids, 72 strains remained for further characterization. In these isolates, PCR identified 11 isolates that appeared to have an unknown Tn*1999* variant. Genomic analysis by whole-genome sequencing (WGS) confirmed a novel Tn*1999* variant, termed Tn*1999.7*, in 10 isolates, and a truncated version, termed ΔTn*1999.7*, in one isolate (Table 1). Tn*1999.7* and ΔTn*1999.7* have not been described before, and no similar sequences could be retrieved from GenBank at the time of writing.

**TABLE 1:** Overview of clinical isolates harboring Tn*1999.7* or ΔTn*1999.7*

Isolate	Isolation mo/yr	Patient ID	Species	ST	OXA-48 plasmid size (bp)	Transposon	Additional plasmid(s) (size in bp)	Genome accession no.
KA14695	10/2016	01	Klebsiella aerogenes	168	71,167	Tn*1999.7*	Untypeable (137,841)	JAKLSS000000000
CF14695	10/2016	01	Citrobacter freundii	22	71,166	Tn*1999.7*	None	JAKLST000000000
EC3239	03/2017	01	Escherichia coli	10	75,519	Tn*1999.7*	Untypeable (98,178), IncFIC (139,536), IncX1 (51,546)	JAKLSU000000000
EC13927	09/2018	02	Escherichia coli	58	71,166	Tn*1999.7*	IncFII (143,810)	JAKLSV000000000
RO13927	09/2018	02	Raoultella ornithinolytica	105	71,166	Tn*1999.7*	None	JAKLSW000000000
CF3626	09/2018	03	Citrobacter freundii	116	71,166	Tn*1999.7*	IncC (104,041), untypeable (90,108), IncFII (54,153)	JAKLSX000000000
CF17067	10/2018	04	Citrobacter freundii	22	63,126	ΔTn*1999.7*	Untypeable (136,836)	JAKLSY000000000
KP17051	12/2018	04	Klebsiella pneumoniae	15	71,166	Tn*1999.7*	IncFIB (109,959), IncR (37,365)	JAKLSZ000000000
CF15807	11/2018	05	Citrobacter freundii	19	71,166	Tn*1999.7*	IncFII (130,575), IncFIB (62,797)	JAKNAE000000000
EC8448	06/2019	06	Escherichia coli	453	71,166	Tn*1999.7*	IncFIB (183,507), p0111 (136,595), IncFII (52,179)	JAKLTA000000000
CF3910	02/2020	07	Citrobacter freundii	19	71,166	Tn*1999.7*	IncFII (130,677), IncFIB (60,937)	JAKLTB000000000

Conventional multilocus sequence typing revealed the same sequence type (ST22 or ST19) in two Citrobacter freundii isolates each. Core genome single nucleotide polymorphism (SNP) analysis of CF14695 and CF17067 (both ST 22) showed four SNPs and a 3-bp insertion difference between the isolates, which were obtained within 24 months of each other. For CF15807 and CF3910, sharing ST19, the difference was three SNPs and they were isolated 15 months apart. For other *Enterobacterales* species, a mutation rate of approximately 3 × 10^−7^ nucleotides per site per year has been reported (8). Assuming similar rates for C. freundii, which has a genome size of ~5 million bp, a genome-wide SNP rate of about 1.5 per year would be expected, not considering mutation-enhancing environments or genetic recombination. Thus, it cannot be excluded that any of the pairs sharing the same ST had a common source. However, with at least 7 of 11 Tn*1999.7*-carrying bacterial isolates being different species, the spread of Tn*1999.7* appears to be plasmid driven rather than clonal.

### Genomic analysis of transposon variants.

**FIG 2:**
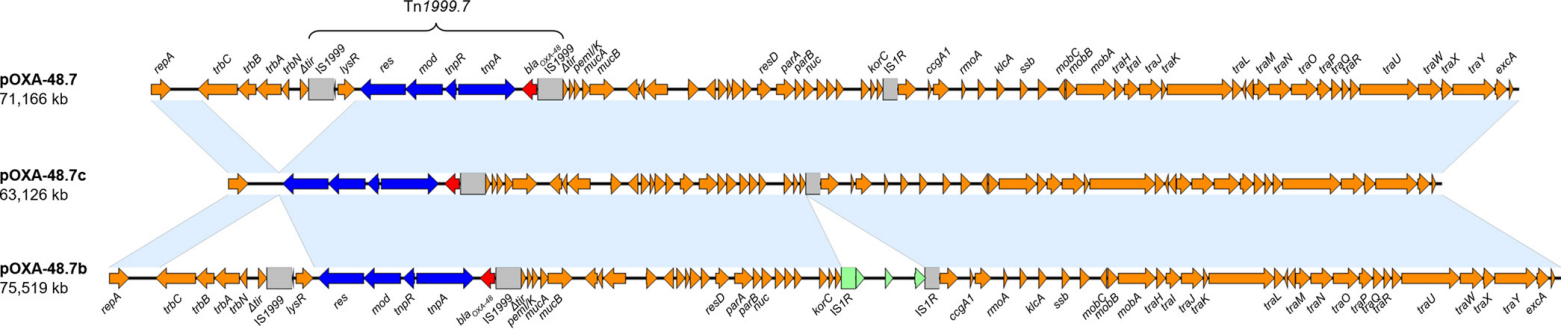
Comparison of the plasmid structures of pOXA-48.7, pOXA-48.7c, and pOXA-48.7b. Red, *bla*_OXA-48_; blue, Tn*7442*; gray, insertion sequences; green, additional insertion sequence in the pOXA-48.7b plasmid backbone; orange, genes of the pOXA-48 plasmid backbone or hypothetical proteins.

Genomic analysis of the WGS hybrid assemblies showed that Tn*1999.7* is equivalent to Tn*1999.1*, with the addition of an 8,349 Tn*3* family transposon (designated Tn*7442* by the Transposon Registry [9]) inserted between *bla*_OXA-48_ and *lysR* (Fig. 1). Tn*7442* appears to be a hybrid transposon, in which the sequence between the resolution site *res1* of *tnpR* and the right inverted repeat is equal to Tn*1*, including the Tn*1* transposase (*tnpA*). Downstream of the resolution site, Tn*7442* comprises a gene coding for a putative resolvase, showing 87% amino acid identity with the Tn*2* resolvase (*tnpR*) (accession no. ALT06231). The resolution site *res1* is a typical recombination site for Tn*3* family transposons (10). In contrast to Tn*1*, which carries *bla*_TEM-2_ as cargo, Tn*7442* appears to carry genes encoding a type III restriction modification (R-M) system. This is composed of two open reading frames (ORFs) showing 99% sequence identity in a REBASE BLAST search with the putative restriction subunit Kgr39ORF30055P (accession no. QLP11302) and the putative DNA methyltransferase subunit M.SmaBWH35ORF26500P (accession no. AVU43264), respectively, with both enzymes sharing the target sequence CCGCAG. From GenBank, plasmid sequences containing Tn*7442* could be retrieved for Klebsiella pneumoniae, Hafnia paralvei, and Citrobacter portucalensis but were from different Inc types and were not associated with *bla*_OXA-48_ (11, 12). The presence of the Tn*7442* insertion in Tn*1999.7* was confirmed by PCR in all isolates. Isolate CF17067 showed a variant of Tn*1999.7*, termed ΔTn*1999.7*, in which the sequence between the left inverted repeat of Tn*7442* and *repA* of the plasmid backbone was deleted (Fig. 1 and 2).

### Analysis of Tn*1999.7*-carrying plasmids.

Tn*1999.7* was located on a 71,166-bp plasmid of the IncL group, termed pOXA-48.7, in 9/11 isolates (Fig. 2). Outside the Tn*1999.7* transposon, the plasmid is highly similar to pOXA-48a (13), but with the insertion of IS*1R* downstream of the *korC* gene as described before for other pOXA-48 variants (7). The plasmid of EC3239 had an additional 4,353-bp insertion upstream of IS*1R*. This insertion encompasses another copy of IS*1R* and three ORFs that match sequences from C. freundii-associated plasmids in GenBank. This plasmid, termed pOXA-48.7b, was 75,519 bp (Table 1 and Fig. 2). The deletion in the plasmid of CF17067 (see the paragraph above) led to a smaller plasmid size of 63,126 bp (Fig. 2). This plasmid was termed pOXA-48.7c.

### Horizontal gene transfer *in vitro*.

To assess the influence of Tn*1999.7* on the transferability of the pOXA-48-type plasmids *in vitro*, liquid mating assays were performed with sodium azide-resistant Escherichia coli J53 and Klebsiella quasipneumoniae PRZ as recipients. Conjugation of the *bla*_OXA-48_-carrying IncL plasmid into J53 was successful for all Tn*1999.7* isolates and into PRZ for all Tn*1999.7* isolates except for Klebsiella aerogenes KA14695. However, the plasmid from the latter strain could be transferred to PRZ via transformation. For C. freundii CF17067, conjugation of the plasmid carrying the truncated variant ΔTn*1999.7* to either recipient strain was unsuccessful. Nevertheless, the plasmid could be transferred by transformation into J53 but not into PRZ. The inability of horizontal gene transfer via conjugation observed in ΔTn*1999.7 in vitro* is most likely caused by the missing *trb* region. Genes of the *trb* operon are essential for assembling the mating pair apparatus. Therefore, the spread of pOXA-48.7c is likely compromised, which is supported by the fact that ΔTn*1999.7* was found in only one patient.

Conjugation rates of the *bla*_OXA-48_-carrying IncL plasmids in J53 were quantified for all Tn*1999.7* isolates and compared to isolates carrying plasmids that share the same plasmid backbone, but carrying *bla*_OXA-48_ on Tn*1999.1*, Tn*1999.2*, a Tn*1999.2* variant, and invTn*1999.*2. For the Tn*1999.7-*carrying isolates, mean conjugation rates ranged from 4.47 × 10^−5^ to 2.03 × 10^−2^, with a mean of means of 3.77 × 10^−3^ (see Fig. S1 in the supplemental material). Mean conjugation rates of the control group ranged from 1.13 × 10^−4^ to 2.81 × 10^−2^, with a mean of means of 6.22 × 10^−3^. These results show no significant difference in the conjugation rates between the two groups. Even though species-specific comparisons between the two groups have only limited significance due to small species groups, the results also indicate no apparent difference in plasmid conjugation rates between the same species that host Tn*1999.7* and other Tn*1999* variants. However, it appears that a higher plasmid number of the donor isolate correlates with a lower conjugation rate in this study. Inhibitory effects of coresiding plasmids are more common than facilitating effects (14). However, a deeper analysis of these correlations is beyond the scope of this study.

Evaluation of plasmid contents of four representative J53 transconjugants (Tc_J53_EC3239, Tc_J53_CF3626, Tc_J53_KP17051, and Tc_J53_EC2013) via long-read sequencing showed that all transconjugants solely contained the pOXA-48-type plasmid. This plasmid could be further conjugated from the J53 transconjugants into another recipient, E. coli DOTN, indicating that no additional plasmids are needed for mobilization.

The antibiotic susceptibility phenotype was determined for the clinical isolates, transconjugants, and transformants (Table S1). Carbapenem MICs of transconjugants/transformants carrying Tn*1999.7* and Tn*1999.2* showed no significant differences.

### Plasmid stability.

**FIG 3:**
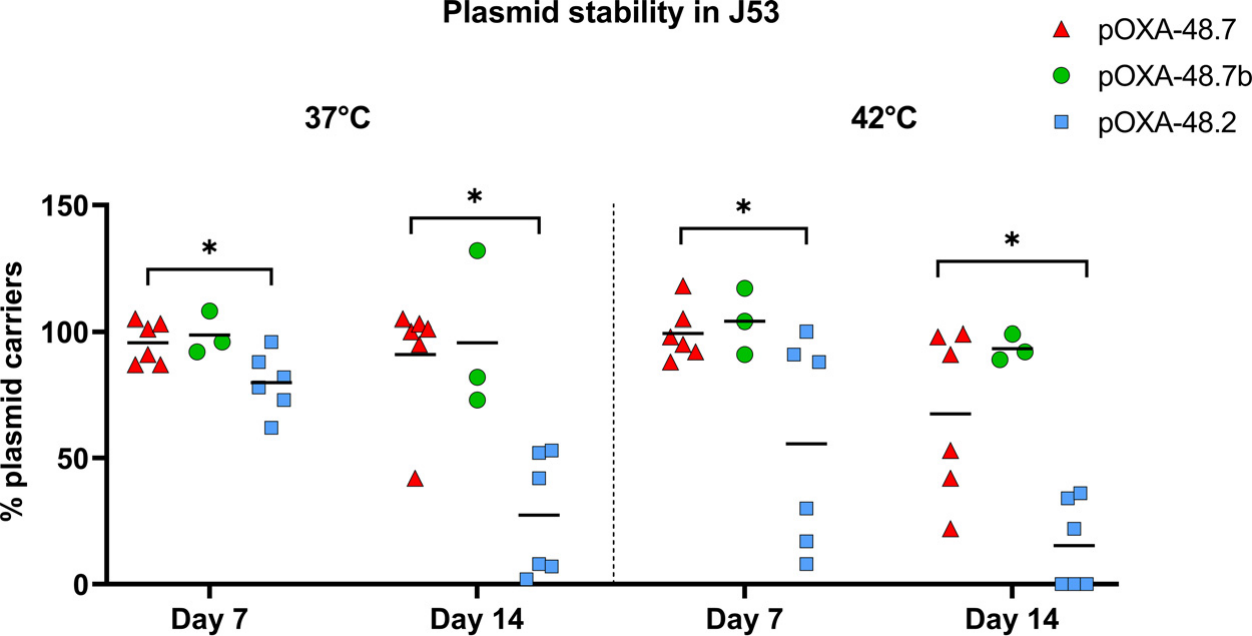
Plasmid stability of the Tn*1999.7*-carrying plasmids pOXA-48.7 and pOXA-48.7b and the Tn*1999.2*-carrying plasmid pOXA-48.2 in J53. Each symbol represents the mean result from one liquid culture plated in duplicate at the respective time point. Asterisks indicate significant differences between pOXA-48.7 and pOXA-48.2.

In Tn*1999.7*, an R-M system was inserted via the transposon Tn*7442.* As effects of R-M systems on plasmid stability have been described (15), plasmid stability was assessed using two different J53 transconjugants carrying pOXA-48.7 (three runs each) or pOXA-48.7b. The stability of these plasmids was compared with that of a J53 transconjugant carrying pOXA-48.2, the most frequently detected Tn*1999.2*-carrying plasmid. At 37°C, pOXA-48.7 was significantly more stable than pOXA48.2 at day 7 (95.7% [SD, 8.1%] versus 79.9% [SD, 11.8%]) and day 14 (90.9% [SD, 24.2%] versus 27.5% [SD, 24.2%]) (Fig. 3). At 42°C, plasmid stability was lower for pOXA-48.2 and pOXA-48.7 at day 14. Still, pOXA-48.7 was significantly more stable at this incubation temperature than pOXA-48.2 (day 7, 99.0% [SD 11.1%] versus 55.6% [SD 41.7%]; day 14, 67.4% [SD 33.0%] versus 15.4% [SD 17.5%]) (Fig. 3). The plasmid stability of pOXA-48.7b was on a similar level to that of pOXA-48.7 at both temperatures.

Restriction-modification systems have been linked to increased plasmid stability (15). This is due to their ability to promote postsegregational killing of bacterial cells missing the plasmid with the R-M system, similar to a toxin-antitoxin system (16). In our study, the potential function of the type III R-M system correlates well with the observation of an increased plasmid stability in the R-M-carrying plasmids pOXA-48.7 and pOXA-48.7b. Nevertheless, definitive conclusions of the function must be drawn with caution as R-M system-related increase of plasmid stability has not been described for type III R-M system yet, but only for type II R-M systems (16). Other potential functions of type III R-M systems (i.e., expression-level changes due to a modified methylome [17]) have not been studied here, which is a limitation that should be addressed in future studies. However, when MICs of antibiotics were compared, no major changes could be observed between J53 transconjugants carrying Tn*1999.7* and those carrying Tn*1999.2*, indicating a similar *bla*_OXA-48_ expression.

In summary, we describe the novel *bla*_OXA-48_-carrying transposon variant Tn*1999.7*, which is associated with increased plasmid stability *in vitro*, presumably due to an inserted R-M system. The increased plasmid stability associated with Tn*1999.7* could contribute to the further spread of OXA-48, which will be revealed by future surveillance data.

## MATERIALS AND METHODS

### Bacterial isolates and antimicrobial susceptibility testing.

*Enterobacterales* isolates carrying *bla*_OXA-48_-like genes were obtained between 2013 and 2020 from clinical samples obtained at the University Hospital Cologne. They were detected during routine diagnostics due to elevated carbapenem MICs, and *bla*_OXA-48_ was verified via the automated PCR system Xpert Carba-R (Cepheid, Sunnyvale, CA, USA) (18). Only one isolate per species for each patient was included in the study. Antibiotic susceptibility was determined by the Micronaut-S broth microdilution panel (Merlin, Bornheim, Germany). Due to a small MIC range for carbapenems in this panel, susceptibility testing was complemented by gradient tests for ertapenem, meropenem, and imipenem (Liofilchem, Roseto degli Abruzzi, Italy).

### PCR-based characterization of *bla*_OXA-48_-harboring isolates.

DNA was extracted using the DNeasy UltraClean microbial kit (Qiagen, Hilden, Germany). Tn*1999.1*, Tn*1999.2*, and invTn*1999.2* were identified by PCR as described before (19). For isolates carrying Tn*1999.7*, the insertion of Tn*7442* was verified by PCR with a long-range high-fidelity polymerase (Phusion; ThermoFisher, MA, USA) with primers Tn_OXA-48-F and TN_TIR (19), resulting in amplicons of 3,512 bp for Tn*1999.1*, 3,508 bp for Tn*1999.2*, and 11,862 bp for Tn*1999.7*.

### Whole-genome sequencing and bioinformatic analyses.

Whole-genome sequencing (WGS) of each sample with Illumina and Oxford Nanopore technology was performed at the Quantitative Biology Center of the University of Tübingen as previously described (20). Sequence data were processed with an in-house pipeline using Snakemake 5.31.1 (21), a python-based script language. Raw reads were quality trimmed with Trimmomatic 0.39 (22), Porechop 0.2.4 (23), and NanoFilt 2.7.1 (24). The quality of the trimmed reads was checked with FastQC 0.11.9 (25), MultiQC 1.9 (26), and NanoPlot 1.32.1 (24). Hybrid assemblies were created with Unicycler 0.4.8 (27), which uses Bowtie2 and Pilon for polishing. Centrifuge 1.0.4_beta (27) was used for species assignment. Annotation and characterization were done by comparing assembled sequences to the databases ResFinder (28), PlasmidFinder (29), and PubMLST (30) and an in-house transposon database with the software ABRicate 1.0.1 (31) and mlst 2.19.0 (32). Core genome single nucleotide polymorphism (SNP) analysis was calculated from raw reads using snippy 4.6.0-1 (33). Annotation from reference plasmids and further analysis was done manually with the software Geneious Prime (Biomatters, Ltd., Auckland, New Zealand). Open reading frames were analyzed via the BLAST function of NCBI (blastx), UniProt (34), ISfinder (35), and the REBASE database (36).

### Analysis of horizontal gene transfer.

Qualitative conjugation was assessed using the sodium azide-resistant strains E. coli J53 and K. quasipneumoniae PRZ as recipient strains (37, 38). Quantitative conjugation was assessed using J53 as the recipient according to a previously published protocol (19) for all Tn*1999.7*-carrying isolates and compared to seven isolates with other Tn*1999* variants. Briefly, liquid mating of clinical isolates and the recipient strains was performed for 2 h, followed by plating on coliform chromogenic agar (CCA) (Carl Roth, Karlsruhe, Germany) containing 20 μg/mL amoxicillin-clavulanate (Hexal, Holzkirchen, Germany) alone or in combination with 100 μg/mL sodium azide. Transconjugation frequency was determined after 48 h of incubation at 37°C by dividing the number of transconjugants (i.e., the number of colonies growing on agar with amoxicillin-clavulanate and sodium azide) by the number of donors (i.e., the number of transconjugants minus the colonies growing on amoxicillin-clavulanate agar without sodium azide).

To assess if J53 transconjugants were able to transfer the plasmid to other recipients, further qualitative conjugation experiments were carried out using representative J53 transconjugants and nalidixic acid-resistant E. coli DOTN as the recipient. CCA supplemented with 128 mg/L nalidixic acid and 20 mg/L amoxicillin-clavulanate was used for selection of DOTN transconjugants.

In case *in vitro* conjugation was unsuccessful, a transformation assay was employed. Plasmid DNA was extracted from clinical isolates utilizing the Plasmid Maxi kit (Qiagen). Transformation of electrocompetent J53 and PRZ bacteria was carried out as described before (39).

### Plasmid stability.

The stability of plasmids carrying Tn*1999.7* was measured using E. coli J53 transconjugants. They were compared to a J53 transconjugant from the above-mentioned isolate collection, with a Tn*1999.2*-carrying plasmid that has the same plasmid backbone as the Tn*1999.7*-carrying plasmids, further referred to as pOXA-48.2. Bacteria were cultured in an antibiotic-free medium for 14 days, and the loss of the resistant population was monitored. A suspension of transconjugants corresponding to a McFarland standard of 1.0 was diluted 1:10^5^, and 10 μL was subsequently added to 4 mL of 1/3 lysogeny broth (LB) (40). Liquid cultures were incubated under agitation (180 rpm) at 37°C, which best reflects the environment in the human gut, and 42°C, to simulate a more challenging environment for the bacteria. Every 24 h, bacterial suspensions were diluted 1:2 × 10^5^ in normal saline, and 10 μL of the dilution was used to inoculate 4 mL of fresh LB. On days 7 and 14, serial dilutions of the liquid cultures were plated on LB agar (LBA) and LBA containing 20 mg/L amoxicillin-clavulanic acid (LBA-AMC), each in duplicate. After overnight culture, the mean number of CFU was determined per duplicate. The ratio of colonies growing on LBA-AMC compared to antibiotic-free LBA was determined to calculate the percentage of bacteria carrying the pOXA-48 plasmid.

### Statistics and graphs.

Plasmid conjugation and stability rates were compared with the Mann-Whitney U test. A *P* value of <0.05 was considered significant. Graphical representations for quantitative conjugation and plasmid stability rates were created with GraphPad Prism 8.4.3. Plasmid graphs were created with Easyfig 2.2.5 (41).

### Ethical approval.

Isolates were collected from routine diagnostics, and only pure cultures of bacterial isolates were investigated. No patient-related data were analyzed. No ethical approval is necessary for this type of study according to the regulations of the University of Cologne.

### Data availability.

The complete nucleotide sequence assemblies of all isolates harboring Tn*1999.7* were deposited publicly in NCBI under BioProject no. PRJNA801874. Accession numbers are listed in Table 1.
