# Supplementary material for: Emergence of Tn1999.7, a New Transposon in blaOXA-48-Harboring Plasmids Associated with Increased Plasmid Stability
Source: Antimicrob Agents Chemother. 2022 Oct 6;66(11):e00787-22. doi: 10.1128/aac.00787-22 (PMC9664867; doi:10.1128/aac.00787-22)
Supplement: Supplemental file 1 — Supplemental material. Download aac.00787-22-s0001.pdf, PDF file, 0.2 MB [file aac.00787-22-s0001.pdf]

Emergence of Tn*1999.7*, a new transposon in *bla*$_{OXA-48}$-harboring plasmids associated with increased plasmid stability

Janko Sattler[a,b], Tsvetan Tsvetkov[a,b], Yvonne Stelzer[c], Sina Schäfer[a,b], Julian Sommer[d], Janina Noster[c], Stephan Göttig[d], Axel Hamprecht[a, b, c #]

[a]Institute for Medical Microbiology, Immunology and Hygiene, University Hospital Cologne and Faculty of Medicine, University of Cologne, Cologne, Germany

[b]DZIF (German Centre for Infection Research), Partner Site Bonn-Cologne, Germany

[c]Institute for Medical Microbiology and Virology, University of Oldenburg and Klinikum Oldenburg, Oldenburg, Germany

[d]Institute for Medical Microbiology and Infection Control, Hospital of Johann Wolfgang Goethe University, Frankfurt, Germany

#Address correspondence to

Axel Hamprecht, University of Oldenburg, Institute for Medical Microbiology and Virology, Brandenburger Str. 19, 26133 Oldenburg, Germany. Tel.: +49 441 403 2160; axel.hamprecht@uol.de

Supplemental material

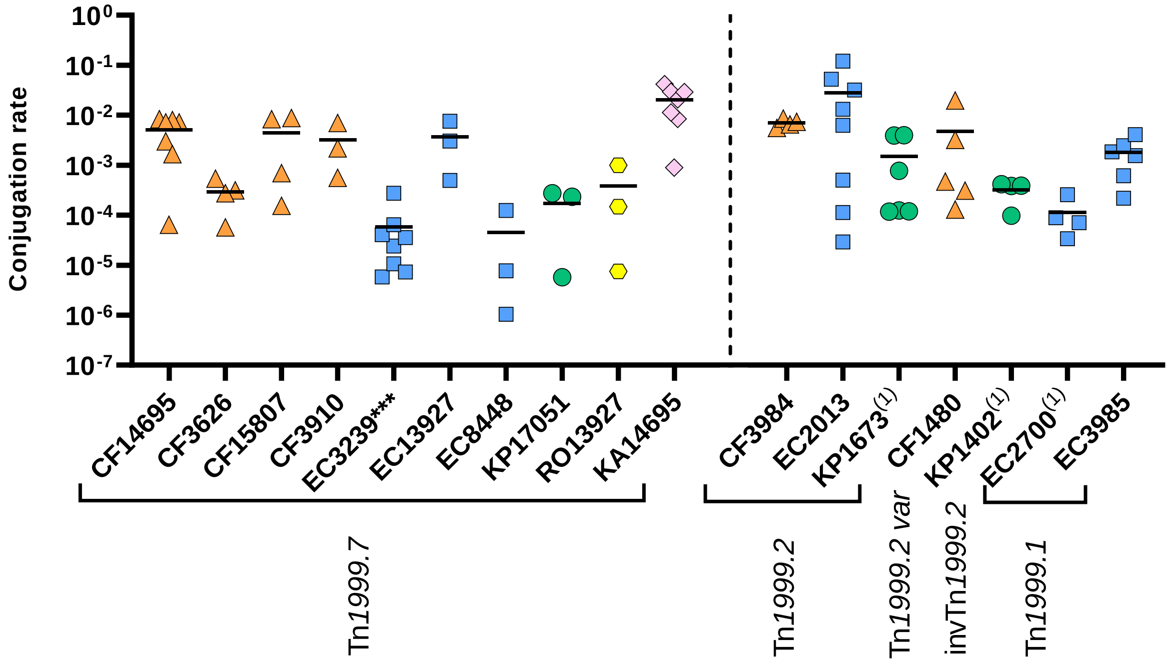

FIG S1: Conjugation rates for IncL plasmids from donor isolates to J53. Each data point represents one conjugation experiment. Color and shape represent donor species. Orange triangle: *C. freundii*, blue square: *E. coli*, green circle: *K. pneumoniae*, yellow hexagon; *R. ornithinolytica*, pink rhombus: *K. aerogenes*. All Tn*1999.7* are located on pOXA-48.7 plasmids, except EC3239*** (pOXA-48.7b).

Table S1: Antibiotic minimal inhibitory concentrations of pOXA-48-harboring clinical isolates, transconjugants (TC) and transformants (TF).

Isolate	TZP	CAZ	ETP	MEM	IPM
EC2013 (Tn*1999.2*)	> 64	< 0.25	8	4	2
CF14695 (Tn*1999.7*)	> 64	1	2	1	4
CF17067 (ΔTn*1999.7*)	> 64	2	4	2	4
J53	2	< 0.25	0.03	0.03	0.25
TC EC2013 in J53	> 64	< 0.25	2	0.25	4
TC CF14695 in J53	> 64	0.5	4	0.5	2
TF CF17067 in J53	> 64	< 0.25	4	1	2
PRZ	4	0.5	0.016	0.03	0.12
TC EC2013 in PRZ	> 64	0.5	4	1	1
TC CF14695 in PRZ	> 64	0.5	2	1	2

TZP = piperacillin-tazobactam, CAZ = ceftazidime, ETP = ertapenem, MEM = meropenem, IPM = imipenem.

References

1. Sommer J, Gerbracht KM, Krause FF, Wild F, Tietgen M, Riedel-Christ S, Sattler J, Hamprecht A, Kempf VAJ, Göttig S. 2021. OXA-484, an OXA-48-type carbapenem-hydrolyzing class D β-lactamase from *Escherichia coli*. Front Microbiol 12:660094–660094.
